# Anthropometry, body composition, early growth and chronic disease risk factors among Zambian adolescents exposed or not to perinatal maternal HIV

**DOI:** 10.1017/S0007114522001775

**Published:** 2023-02-28

**Authors:** Suzanne Filteau, Lackson Kasonka, Jonathan C. K. Wells, Grace Munthali, Molly Chisenga, Andrea Mary Rehman

**Affiliations:** 1 Faculty of Epidemiology and Population Health, London School of Hygiene and Tropical Medicine, Keppel Street, London WC1E7HT, UK; 2 University Teaching Hospital – Women and Newborn, Lusaka, Zambia; 3 Institute of Child Health, University College London, London, UK; 4 National Institute for Scientific and Industrial Research, Lusaka, Zambia

**Keywords:** HIV, Growth trajectory, Body composition, Chronic disease risk, Adolescent, HbA1c, Cohort

## Abstract

Early life exposures and growth patterns may affect long-term risk of chronic non-communicable diseases (NCD). We followed up in adolescence two Zambian cohorts (*n* 322) recruited in infancy to investigate how two early exposures – maternal HIV exposure without HIV infection (HEU) and early growth profile – were associated with later anthropometry, body composition, blood lipids, Hb and HbA1c, blood pressure and grip strength. Although in analyses controlled for age and sex, HEU children were thinner, but not shorter, than HIV-unexposed, uninfected (HUU) children, with further control for socio-demographic factors, these differences were not significant. HEU children had higher HDL-cholesterol than HUU children and marginally lower HbA1c but no other biochemical or clinical differences. We identified three early growth profiles – adequate growth, declining and malnourished – which tracked into adolescence when differences in anthropometry and body fat were still seen. In adolescence, the early malnourished group, compared with the adequate group, had lower blood TAG and higher HDL, lower grip strength (difference: −1·87 kg, 95 % CI −3·47, −0·27; *P* = 0·02) and higher HbA1c (difference: 0·5 %, 95 % CI 0·2, 0·9; *P* = 0·005). Lower grip strength and higher HbA1c suggest the early malnourished children could be at increased risk of NCD in later life. Including early growth profile in analyses of HIV exposure reduced the associations between HIV and outcomes. The results suggest that perinatal HIV exposure may have no long-term effects unless accompanied by poor early growth. Reducing the risk of young child malnutrition may lessen children’s risk of later NCD.

The prevalence of chronic non-communicable diseases (NCD) is rising globally, including in low- and middle-income countries^(1)^. Overweight and obesity, which are also increasing in prevalence, are important risk factors for NCD, but there is evidence from Africa that some NCD, notably diabetes, may frequently occur in the absence of overweight and at younger ages than seen in high-income countries^(2)^. Environmental factors, both concurrent and earlier in life, are likely to be important contributors to the different phenotypes of NCD in Africa compared with those in high-income countries.

Severe infectious diseases such as malaria, HIV and tuberculosis are common in Africa. Malaria both earlier in life and concurrently among Ugandan children aged about 10 years was associated with altered blood lipid profiles^(3)^. HIV infection in adults can increase the risk of some NCD^(4–6)^ and people who start antiretroviral therapy (ART) with advanced disease, as indicated by a low CD4 count, may be especially at high risk^(7)^. Advanced HIV can also lead to weight loss and we have shown that prior malnutrition due mainly to HIV or tuberculosis was associated with later increased risk of low insulin production among Tanzanian adults^(8)^.

Although being HIV-infected seems to increase the risk of NCD, there is limited information as to whether there are similar risks of perinatal exposure to maternal HIV in children who do not themselves become infected, that is, who are HIV-exposed, uninfected (HEU). For these children, some effects of HIV exposure may be indirect, rather than direct causes of the virus. Figure 1 shows a conceptual framework linking exposure to maternal HIV, socio-demographic variables and early growth pattern to the outcome of NCD risk. Children of HIV-infected mothers may be born at lower birth weight than HIV-unexposed, uninfected (HUU) children^(9,10)^ which may increase their NCD risk^(11)^. The lower birth weight could result from not only virus-induced factors such as inflammation^(12)^ but also socio-economic factors such as HIV-infected parents being less able to work with consequences for household food security and nutrition^(13)^. Demographic factors may also interact with HIV and socio-economic factors over time, for example, HIV-infected parents may die, leaving their children as single or double orphans with consequent economic and other risks for nutrition and health. The long-term NCD risk appears to occur with malnutrition in childhood as well as prenatally, as evidenced by studies showing that exposure to famine during childhood was associated with increased adult diabetes in the Netherlands^(14)^ and China^(15)^.

**Fig. 1.**
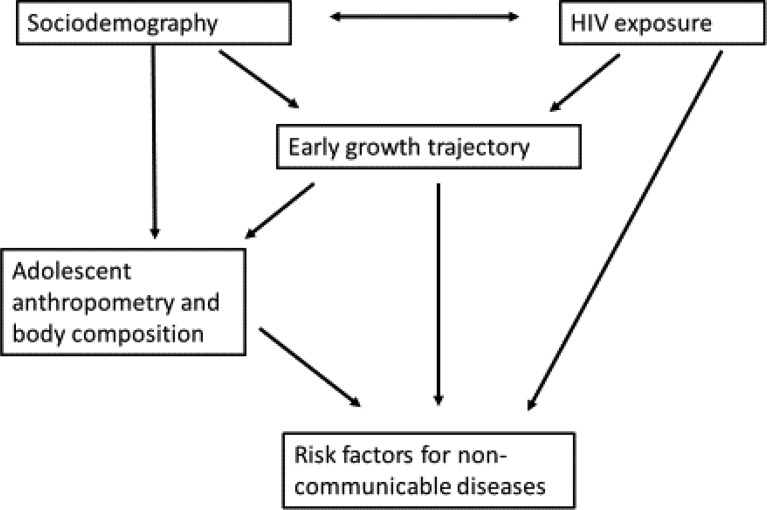
Conceptual framework linking HIV exposure and early growth trajectory to later risk factors for non-communicable diseases.

Although catch-up growth before aged 2 years after being born low birth weight appears to carry little risk, later fast growth may increase NCD risk^(16,17)^. Fast childhood weight gain is becoming increasingly common in countries undergoing the nutrition transition^(18)^, although it may be less common in children of the lowest socio-economic status (SES). While overt NCD typically emerge in adult life, many studies have linked markers of fetal undernutrition with markers of poorer cardiometabolic health during childhood, such as raised blood pressure, insulin resistance and dyslipidaemia that may track into adulthood^(19,20)^.

We have studied two cohorts of Zambian children whom we recruited for prior studies related to nutrition and HIV in order to investigate long-term effects of perinatal HIV exposure or early linear and ponderal growth trajectory on anthropometry, body composition and biomarkers for NCD in early adolescence.

## Methods

### Design

This follow-up study, with field work conducted from 2018 to 2019, was an analysis of children from previously followed up cohorts. Our primary exposures were perinatal exposure to maternal HIV and growth trajectory profiles in early life.

### Participants

There were two separate cohorts of children: one previously recruited for a randomised controlled nutrition trial and the other for an observational cohort study. Both studies were conducted by the same research team and recruited from the same catchment area of Lusaka, Zambia. Most of these children had been previously followed up in 2014^(21)^.

For the Breastfeeding and Postpartum Health (BFPH) longitudinal cohort study^(9)^, HIV-infected and uninfected mothers of the children were recruited when pregnant and children were born between 2001 and 2004. Detailed information on maternal and infant health, infant feeding and infant growth was collected until age 16 weeks. HIV status of all mothers was known through antenatal testing at the local government clinic. Children’s HIV status was not assessed in the original BFPH study. At the time of the study, the only ART regimen available for prevention of mother-to-child transmission in the area was perinatal nevirapine to both mother and infant. ART was not available for the women themselves. Recruited HIV-infected women were slightly older and less likely to be primiparous than HIV-uninfected women, and infants of the HIV-infected women were born at about 100 g lower weight^(9)^.

For the Chilenje Infant Growth, Nutrition and Infection Study (CIGNIS) randomised controlled trial comparing two locally made complementary foods, children born between 2005 and 2007 were recruited at age 6 months and participated until they were 18 months^(22)^. At the time of the study, perinatal nevirapine was the local regimen for prevention of mother-to-child transmission. ART was available only for adults with CD4 count < 200 cells/µl until towards the end of the study when the cut-off was changed to < 350 cells/µl; only five of the CIGNIS children’s mothers were on any ART. Agreement to HIV testing of children by antibodies at 18 months, the only test available locally throughout most of the trial, was an inclusion criterion of the study. Children who died or defaulted before 18 months were not tested for HIV. Knowledge of maternal HIV status was not required, although antenatal HIV status from routine government health services was known for most of the women. HIV-infected mothers were older than HIV-uninfected mothers, were of lower education and were more likely to be in the lowest tercile of an asset index. HIV-infected mothers were less likely to initiate breast-feeding and stopped earlier compared with HIV-uninfected mothers^(23)^. There were no differences in linear growth, the trial primary outcome, between the diet treatment groups^(22)^.

### HIV status and exposure

Children of HIV-uninfected mothers who had themselves never been tested were considered HUU. Children of HIV-infected mothers whose own HIV status was missing were included as HEU since we expected that, by adolescence, they would have begun to show symptoms on clinical examination if they were perinatally HIV-infected. Children whose mother’s HIV status was unknown were coded as unknown HIV exposure. Only seven children were HIV-infected at the recent follow-up; they contributed to the determination of early growth trajectories but were not included in analyses of adolescent outcomes since it is already established that HIV-infected children often grow poorly. Furthermore, because they were few in number we lacked statistical power to include them as a comparison group. We therefore compared HUU against HEU.

### Ethical approval

This study was conducted according to the guidelines laid down in the Declaration of Helsinki and all procedures involving human participants were approved by the University of Zambia Biomedical Research Ethics Committee and the London School of Hygiene and Tropical Medicine ethics committee. Written consent was obtained from carers for participants under 18 years old and from the participants themselves if over 18 years. Written assent was also obtained from all under-age children. If a reason for medical intervention was found on clinical examination, children were referred to the local government services on the same site as the clinic.

### Assessments at study visits

Children and parents or guardians were invited to the University Teaching Hospital research clinic for a single visit. Questionnaires were used to collect demographic, socio-economic and morbidity history data, and children were given a clinical examination. We asked girls if they had started menstruating and we examined boys for Tanner stage.

Anthropometry data – weight, height, mid-upper arm circumference, waist and hip circumferences, triceps, subscapular and suprailiac skinfolds – were collected by research nurses trained and experienced in anthropometry. The child’s right side was used for mid-upper arm circumference and triceps skinfold. Blood pressure was measured using an automatic Omron IP20 sphygmomanometer. Grip strength for both hands was measured using a Takei GRIP-D dynamometer. The measurements, excluding body composition, were taken twice and analyses used the mean for most variables but the maximum for grip strength.

Venous blood samples were collected for TAG and total, HDL- and LDL-cholesterol. Blood lipids were measured using enzymatic assay kits from Pointe Scientific. Finger-prick blood samples were used for measurement of Hb and HbA1c using handheld instruments from Hemocue; due to problems with equipment or supplies, some children did not have these measurements. HbA1c was the only feasible method for assessing diabetes risk in the study because it was not possible to have children come to the clinic fasting or to conduct glucose tolerance tests. Mild anaemia was defined as Hb ≥ 80 g/l and < 120 g/l and severe anaemia as Hb < 80 g/l. It is unclear what level of HbA1c should be considered high in this population since preliminary analysis using a cut-off of > 5·43 %, which is the 90^th^ percentile for American children aged 10–14 years^(24)^, found an unlikely percentage (82 %) of the cohort with high HbA1c, possibly due to differences in genetic ancestry^(25)^. We chose to use a common adult cut-off for diabetes, HbA1c ≥ 6·5 %, for internal comparisons, while recognising the limited ability to predict diabetes risk.

Body composition was measured using three independent methods: bioelectrical impedance (BIA) using a Tanita BC418 instrumentation, air displacement plethysmography (ADP) using a BodPod (Life Measurement) and ^2^H (D_2_O) dilution according to standard methods^(26)^. For BIA and ADP, fat mass and fat-free mass (FFM) were obtained using in-built manufacturers’ equations. For D_2_O dilution, a baseline saliva sample was collected from participants at least 2 h after their last meal. Each participant then received an oral dose (0·1 g/kg body weight) of D_2_O (99·8 % atom excess, Cambridge Isotope Laboratories). Two end-point saliva samples were collected at 3 and 4 h after D_2_O dose ingestion; if they agreed within 3 mg/kg, indicating equilibration by 3 h, the average was used; if not, the 4-h sample was reanalysed as the duplicate. Saliva samples were stored in plastic saliva vials at –20°C until analysis for D_2_O abundance using a Fourier transform IR spectrometer (Agilent Technologies, model 4500s). The enrichment was calculated by subtracting the value of the baseline sample from the value of the post-dose sample. The calculated D_2_O enrichment was then used in the calculation of body composition, using published values for FFM hydration to convert body water to FFM^(27)^. Fat mass was calculated by difference of weight and FFM. For all body composition measures, we used in analyses fat mass index (FMI) and fat-free mass index (FFMI) which were calculated by dividing kgs fat or FFM by height squared, analogous to BMI.

### Data management and statistical analyses

Data were collected using the RedCap system and imported into Stata 16.1 for analysis. Height-for-age and BMI-for-age Z scores were calculated using the WHO standards in the Stata zanthro command. Principal component analysis was used to create a SES score from questionnaire data on family assets – car, bicycle, radio, television, refrigerator, mobile phone, livestock and poultry. The SES score was divided into terciles of low, middle and high SES. The principal component analysis for SES was based on a larger group (*n* 514) of families from a wider cross-section of Lusaka neighbourhoods since we were conducting a related study in parallel^(28)^. We collected data on both maternal and paternal education and occupation but present only maternal since a large proportion of paternal data was missing.

Preliminary analyses comparing FMI from the three measures of body composition (BIA, ADP and ^2^H dilution) found that correlation coefficients among the measures ranged from 0·83 to 0·92, all with *P* < 0·001. FMI was slightly lower for ADP (and consequently FFMI slightly higher since they sum to BMI) but the differences were small; FMI was 3·98 kg/m^2^ (sd 3·22) by ADP, 4·39 kg/m^2^ (sd 2·67) by BIA and 4·47 kg/m^2^ (sd 2·91) by D_2_O. This suggested that the three body composition methods, though having different underlying assumptions and potential sources of error, were in general agreement. Moreover, as demonstrated previously^(29)^, an aggregate value for body composition obtained from several methods is more accurate than values from individual methods, as the error associated with each technique tends to cancel out. Therefore, we used the FMI and FFMI outcomes in analyses as the arithmetic means of results from the three methods^(29)^. Although there was a small amount of missing data for body composition by each method alone (1 for D_2_O, 2 for ADP, 19 for BIA), the combined FMI and FFMI outcomes had no missing data.

We conducted two sets of analyses of the same outcome variables with different exposure variables. The first exposure was perinatal exposure to maternal HIV for which we compared HEU children with HUU children. The second exposure was early growth trajectory profile. To identify these, we used latent class structural equation models, the *gsem* command and weight-for-age and length-for-age Z scores from three time points: birth (only weight available in CIGNIS), infancy (4 months in BFPH, 6 months in CIGNIS) and early childhood (∼3 years in BFPH, 18 months in CIGNIS). All data available, including from children who died or were lost to follow-up, were included in the determination of growth trajectory profile. To determine the optimal number of profiles which fitted the data best, we used the Bayesian information criteria (lower values are better), entropy values (values closer to 1 are better), predicted posterior probabilities (considered the certainty that a given participant was a member in their particular allocated profile) and ensured sample sizes in each group were a minimum of sixty-two individuals (5 % of the sample size available to determine latent class grouping).

Outcome variables compared between our exposure groups were anthropometry, FMI, FFMI, blood lipids, blood pressure, Hb, HbA1c and grip strength. The primary analyses, using linear regression to generate mean differences and 95 % CI, were controlled for age and sex. We then conducted multivariable analyses adjusting for factors which either differed among HIV exposure groups in order to separate HIV exposure itself from socio-demographic factors associated with exposure or were associated with loss to follow-up since original recruitment in order to control for the survivor or other follow-up bias. Pubertal stage was not included as a covariate in analyses since it was collinear with age. To analyse all children’s records, including the 26 who were missing HIV exposure information, the 87 missing HbA1c and the 146 missing Hb plus a few other variables with small amounts of missing data, we used multiple imputation with chained equations to generate ten multiple imputation data sets including exposures and all outcomes in the imputation model. We used overall Wald tests to determine the association between exposures and outcomes with a significance level of 0·05 and no correction for multiple testing.

In exploratory analysis and without formally fitting mediation models, we wished to investigate whether there was any evidence that early growth trajectory mediated the relationship between HIV exposure and outcomes. Therefore, for outcomes associated with HIV exposure in age- and sex-controlled analyses, we added an early growth profile to the regression models to determine whether associations with HIV exposure were modified by the inclusion of early growth profile. We chose to include outcomes where the relationship between HIV exposure and outcome had *P* < 0·05 in the age- and sex-controlled analyses, rather than those in the multivariable analyses, because the multiple associations between maternal HIV, early growth trajectory and socio-demographic variables meant that inclusion of the latter would be over-controlling.

### Sample size

The sample size was dictated by the number of cohorts who were available at follow-up (*n* 322). This number of children provided 90 % power to detect outcome differences of effect size 0·36 between the two groups of HIV exposure and 0·44 between pair-wise comparisons of the three early growth trajectory profiles. In order to have adequate statistical power when fitting structural equation models, the sample size of 322 individuals meets recommendations of at least twenty participants per explanatory variable^(30)^.

## Results

**Fig. 2.**
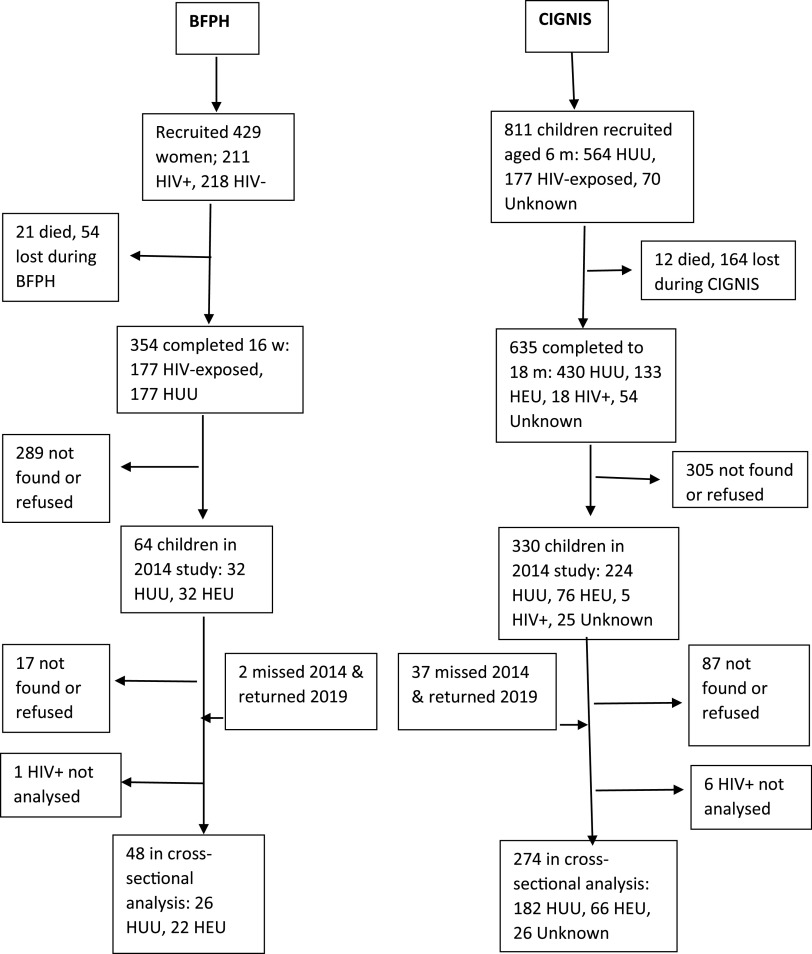
Flow chart of study participants. BFPH, Breastfeeding and Postpartum Health; CIGNIS, Chilenje Infant Growth, Nutrition and Infection Study; HUU, HIV-unexposed, uninfected; HEU, HIV-exposed, uninfected.

Figure 2 shows that at this follow-up, 322 HIV-uninfected children and adolescents were available from the original BFPH (*n* 48) and CIGNIS (*n* 274) studies. Seven HIV-infected children were recruited but not analysed for their follow-up data. Although dropout was high from both studies, especially from BFPH for whom their mothers were recruited 17–20 years ago, BFPH participants studied in 2019 did not differ from those in the original cohort who were not followed up in terms of sex ratio, birth weight, HIV exposure or socio-demographic variables (online Supplementary Table S1). The CIGNIS children followed up did not differ from those not followed up in sex ratio, birth weight or HIV exposure but were of higher SES and their mothers were more likely to have been married and employed.

### Associations of outcomes with HIV exposure

HIV status and exposure were known for all participants except twenty-six from the CIGNIS study. The proportion of HIV-exposed participants was similar within each cohort over time. All groups were evenly divided between boys and girls (Table 1). The HIV-unknown group was slightly younger and boys were at earlier pubertal stage than the other groups, likely because there were no BFPH children in this group. Socio-economic tercile and maternal occupation did not differ among groups, but fewer mothers of HEU children were married and they tended to have less education than mothers of HUU or HIV-unknown children.

**Table 1.** Characteristics of children according to HIV exposure (Numbers and percentages; mean values and standard deviations)

	HIV-unexposed		HIV-exposed, uninfected		HIV-unknown exposure		
	*n*	%	*n*	%	*n*	%	*P* *
*n*	208		88		26		
Sex, male	103	50	40	45	14	54	0·7
Age (years)							
Mean	12·8		13·3		11·8		< 0·001
sd	1·5		2·0		1·0		
Girls started menstruating†	55/104	53	27/48	56	4/12	42	0·66
Boys’ Tanner stage†							
1	3/102	3	0/40	0	0/14	0	0·05
2	37/102	36	10/40	25	9/14	64	
3	31/102	30	10/40	25	4/14	29	
4	27/102	26	14/40	35	1/14	7	
5	4/102	4	6/40	15	0/14	0	
Mother’s marital status							
Married/cohabiting	153	74	49	56	22	85	0·004
Single	19	9	11	13	1	4	
Widowed	18	9	14	16	0		
Divorced	14	7	9	10	2	8	
Unknown	4	2	5	6	1	4	
Mother’s education,							
Primary or less	24	12	16	18	5	19	0·006
Some secondary	40	19	32	36	3	12	
Completed secondary	53	25	17	19	7	27	
College, university	88	42	18	20	10	38	
Unknown	3	1	5	6	1	4	
Mother’s occupation							
Employed	154	74	54	61	17	65	0·36
Housewife	36	17	21	24	5	19	
Unemployed/student	6	3	6	7	2	8	
Other/unknown	12	6	7	8	2	8	
Socio-economic tertiles‡							
Low	90	43	51	58	12	46	0·11
Medium	26	13	11	13	3	12	
High	92	44	26	30	11	42	

* *P* values are from ANOVA for continuous variables and *χ* ^2^ for categorical variables.

† Menstruation missing for 1 HIV-unexposed girl and Tanner stage for 1 HIV-unexposed boy.

‡ Calculated using principal components analysis from a list of assets terciles included another group of children followed at the same time for a different study which is why terciles are imbalanced.

Table 2 shows marginal mean differences between HIV exposure groups and anthropometry, body composition and grip strength using the imputed data set; crude means without imputation are shown in online Supplementary Table S2. Almost all differences were negative, that is, the HEU children were smaller, and a few outcomes (hip circumference, triceps and subscapular skinfolds, FMI) were different at *P* < 0·05 from HUU children in analyses controlled for age and sex. However, when further controlled for factors associated with HIV exposure or loss from the initial cohort (children of more educated or married mothers or in the higher socio-economic terciles tended to be larger), there were no significant associations of outcomes with HIV exposure. Girls did not differ from boys in height but had higher BMI and indicators of body fat (data not shown).

**Table 2.** Association of HIV exposure and status with anthropometry, body composition and grip strength using data from multiple imputation*,† (Odds ratios and 95 % confidence intervals)

	Imputed marginal means		Coefficient adjusted for age and sex			Multivariable coefficient		
Outcome	Means	95 % CI§	*β*	95 % CI	*P*	*β*	95 % CI||	*P*
Height (cm)								
HUU	152·3	151·1, 153·5	Ref			Ref		
HEU	152·3	150·7, 154·0	–0·01	–1·71, 1·69	0·99	0·84	–0·90, 2·59	0·34
Height-for-age Z								
HUU	–0·27	–0·39, −0·14	Ref			Ref		
HEU	–0·28	–0·45, −0·11	–0·01	–0·23, 0·20	0·89	0·09	–0·14, 0·32	0·44
Weight (kg)								
HUU	45·7	44·0, 47·4	Ref			Ref		
HEU	43·5	41·5, 45·5	–2·18	–4·59, 0·22	0·08	–0·60	–3·02, 1·81	0·62
BMI (kg/m^2^)								
HUU	19·5	18·9, 20·1	Ref			Ref		
HEU	18·6	17·9, 19·3	–0·91	–1·80, −0·02	0·05	–0·41	–1·31, 0·49	0·37
BMI-for-age Z								
HUU	0·04	–0·14, 0·23	Ref			Ref		
HEU	–0·22	–0·46, 0·03	–0·26	–0·57, 0·05	0·1	–0·07	–0·38, 0·25	0·67
MUAC (cm)								
HUU	23·7	23·2, 24·3	Ref			Ref		
HEU	23·0	22·4, 23·6	–0·8	–1·6, 0·0	0·06	–0·3	–1·1, 0·6	0·56
Hip circumference (cm)								
HUU	83·5	82·0, 85·1	Ref			Ref		
HEU	81·2	79·4, 83·0	–2·3	–4·5, −0·1	0·04	–0·9	–3·1, 1·4	0·45
Waist circumference (cm)								
HUU	65·8	64·6, 67·1	Ref			Ref		
HEU	64·3	62·8, 65·9	–1·5	–3·4, 0·5	0·13	–0·5	–2·5, 1·4	0·59
Triceps skinfold (mm)								
HUU	13·0	12·1, 13·8	Ref			Ref		
HEU	11·4	10·3, 12·5	–1·5	–2·9, −0·2	0·03	–0·8	–2·2, 0·6	0·26
Subscapular skinfold (mm)								
HUU	11·0	10·2, 11·8	Ref			Ref		
HEU	9·5	8·7, 10·4	–1·4	–2·6, −0·3	0·02	–0·8	–1·9, 0·4	0·19
Suprailiac skinfold (mm)								
HUU	10·2	9·3, 11·1	Ref			Ref		
HEU	9·0	7·9, 10·1	–1·2	–2·5, 0·2	0·1	–0·5	–1·9, 0·9	0·5
Fat mass index (kg/m^2^)‡								
HUU	4·5	4·1, 4·9	Ref			Ref		
HEU	3·8	3·4, 4·3	–0·62	–1·20, −0·03	0·04	–0·37	–0·97, 0·22	0·22
Fat-free mass index (kg/m^2^)‡								
HUU	14·9	14·7, 15·2	Ref			Ref		
HEU	14·7	14·4, 15·1	–0·22	–0·66, 0·23	0·34	0·07	–0·40, 0·55	0·76
Grip strength (kg)								
HUU	21·9	21·1, 22·6	Ref			Ref		
HEU	22·0	21·0, 23·0	0·2	–1·0, 1·3	0·27	0·5	–0·8, 1·7	0·47

* HEU, HIV-exposed, uninfected; HUU, HIV-unexposed, uninfected; MUAC, mid-upper arm circumference.

† There were 227 children in the HUU group and ninety-five in the HEU group. Numbers in analyses differed by imputation data set.

‡ Mean body composition combining results from bioelectrical impedance, air displacement plethysmography and ^2^H dilution methods. Indices are fat or fat-free mass in kg divided by height in metre squared.

§ Marginal means controlling for age and sex; marginal means from multivariable differed from these only modestly and are not shown.

|| Multivariable coefficients represent the difference from the HUU group, adjusted for age, sex, maternal education, maternal marital status and socio-economic tercile.

Table 3 shows marginal mean differences in biochemical data according to child HIV exposure. Few differences were seen except that, in both age- and sex-controlled and fully controlled analyses, HEU children had higher blood HDL, and in the fully adjusted analysis, HEU children had borderline lower HbA1c than HUU children. Based on the imputed data, mild anaemia (Hb between 80 and 120 g/l) was present in 27 % of HUU and 28 % of HEU children and severe anaemia (Hb < 80 g/l) was present in 5 % of HUU and 2 % of HEU children (*χ* ^2^ *P* = 0·001). HbA1c was ≥ 6·5 % for 53 % of HUU and 47 % of HEU children.

**Table 3.** Association of HIV exposure and infection with clinical variables using data from multiple imputation*,† (Odds ratios and 95 % confidence intervals)

	Imputed marginal means		Difference adjusted for age and sex			Multivariable coefficient		
Outcome	Means	95 % CI‡	*β*	95 % CI	*P*	*β*	95 % CI§	*P*
Hb (g/l)								
HUU	106	102, 109	Ref			Ref		
HEU	103	98, 108	–2·6	–8·7, 3·5	0·4	–2·4	–8·9, 4·1	0·47
HbA1c (%)								
HUU	5·9	5·7, 6·0	Ref			Ref		
HEU	5·7	5·5, 5·8	–0·2	–0·5, 0·1	0·12	–0·3	–0·5, 0·0	0·05
Systolic blood pressure (mmHg)								
HUU	105	103, 106	Ref			Ref		
HEU	106	103, 108	1·0	–1·7, 3·7	0·46	1·1	–1·6, 3·8	0·42
Diastolic blood pressure (mmHg)								
HUU	66	65, 66	Ref			Ref		
HEU	66	64, 67	0·2	–1·6, 2·1	0·8	0·0	–1·8, 1·8	0·98
Blood lipids								
TAG (mmol/l)								
HUU	0·84	0·78, 0·90	Ref			Ref		
HEU	0·79	0·71, 0·88	–0·05	–0·16, 0·06	0·4	–0·02	–0·13, 0·09	0·74
Cholesterol (mmol/l)								
HUU	3·83	3·71, 3·94	Ref			Ref		
HEU	3·87	3·72, 4·02	0·04	–0·15, 0·23	0·69	0·11	–0·09, 0·30	0·26
HDL-cholesterol (mmol/l)								
HUU	1·09	1·05, 1·13	Ref			Ref		
HEU	1·16	1·11, 1·22	0·07	0·00, 0·14	0·04	0·08	0·01, 0·15	0·03
LDL-cholesterol (mmol/l)								
HUU	2·35	2·26, 2·44	Ref			Ref		
HEU	2·34	2·22, 2·47	–0·01	–0·16, 0·15	0·94	0·05	–0·11, 0·22	0·51

* HEU, HIV-exposed, uninfected; HUU, HIV-unexposed, uninfected.

† There were 227 children in the HUU group and ninety-five in the HEU group. Numbers in analyses differed by imputation data set.

‡ Marginal means controlling for age and sex; marginal means from multivariable differed from these only modestly and are not shown.

§ Multivariable coefficients represent the difference from the HUU group, adjusted for age, sex, maternal education, maternal marital status and socio-economic tercile.

### Associations of outcomes with latent class growth profiles

**Fig. 3.**
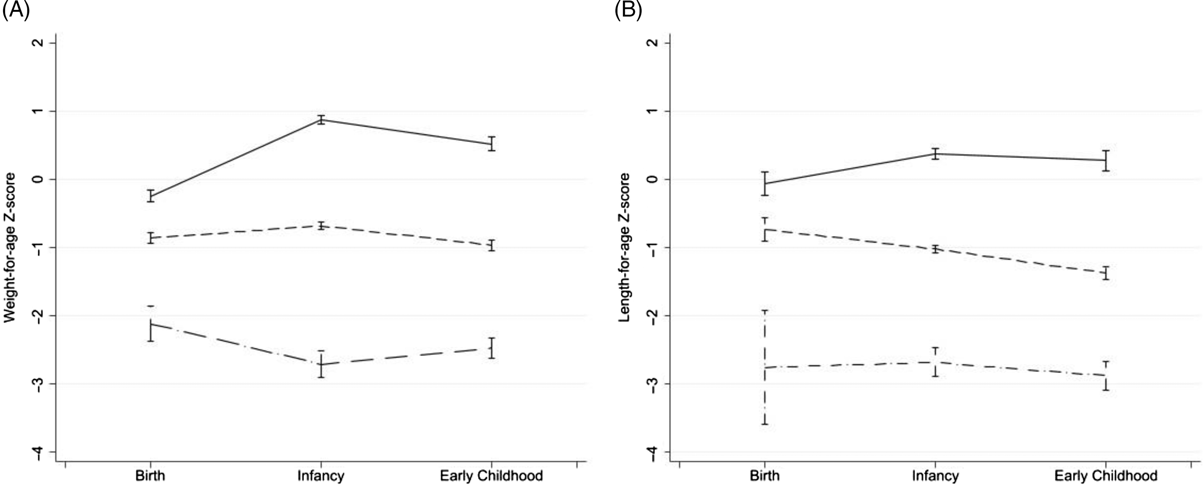
Early growth trajectory profiles determined by latent class analysis. (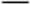) Adequate growth; (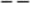), declining; (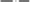), malnourished.

Three latent class profiles were determined from analysis of the early growth data; we call these adequate growth, declining and malnourished (Fig. 3). The adequate growth group (454 children from the combined original data sets) gained considerably in weight-for-age Z score and somewhat in length-for-age Z score during early life, the decliners (683 children) had fairly stable weight-for-age Z score but declining length-for-age Z score and the malnourished group (104 children) had mean weight-for-age Z score and length-for-age Z score < –2 at all time points. More CIGNIS than BFPH children were in the declining (60 % *v*. 46 %) or malnourished (9·5 % *v*. 6·3 %) groups. Unsurprisingly, children who died during the original studies were more likely to be in the declining class (67 % *v*. 54 % for those who completed the original study and 59 % for those lost to follow-up) or the malnourished class (21 % *v*. 7·7 % among completers and 9·7 % among those lost). More children of HIV-infected mothers than of HIV-uninfected or HIV-unknown mothers (12 % *v*. 7 % and 9 %) were in the malnourished group. Child HIV status was mostly unknown during the ages of data in the latent variable analysis but we know few children overall were HIV-infected themselves. Child sex was not associated with the latent class.

**Fig. 4.**
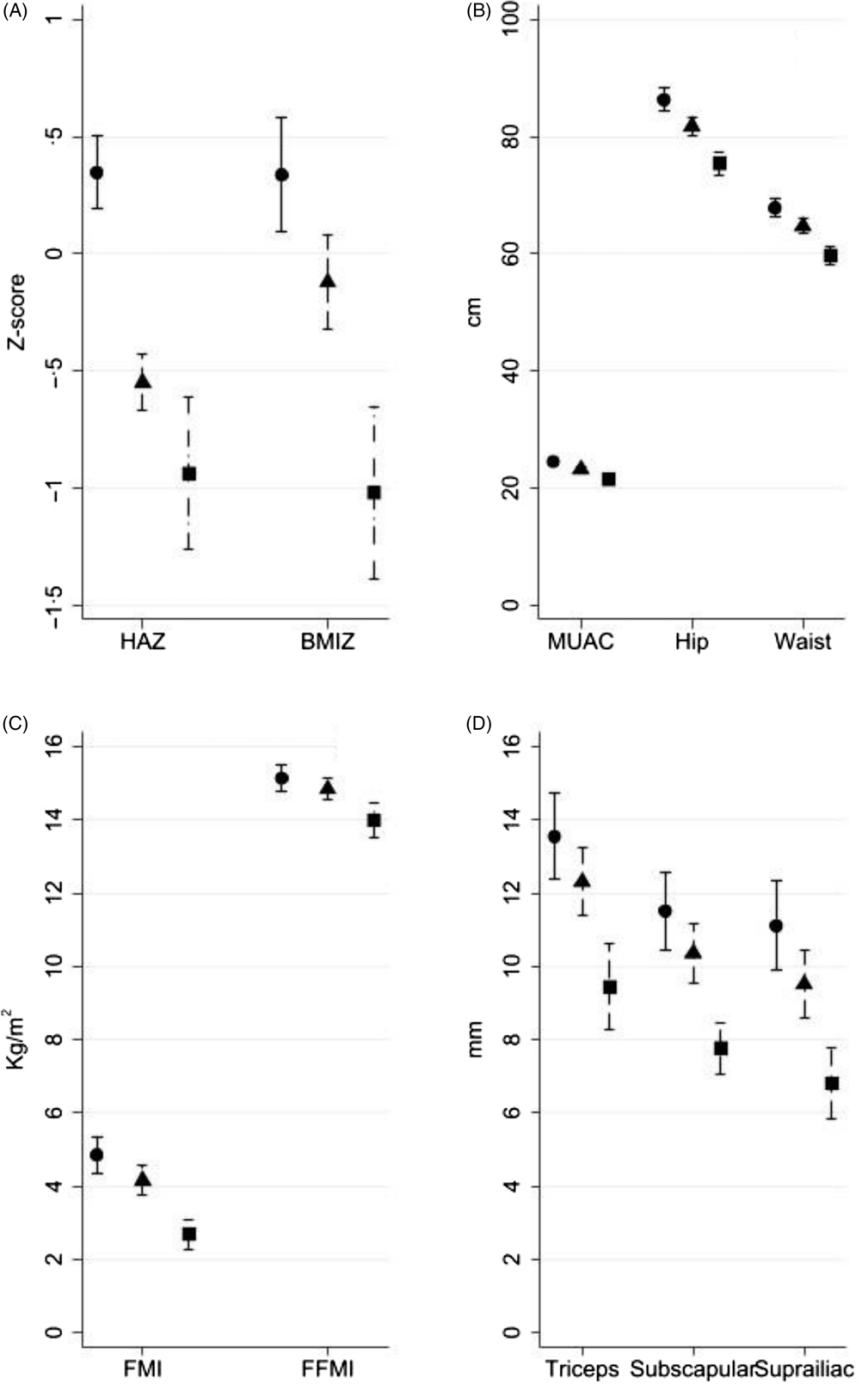
Anthropometry at follow-up according to early growth trajectory profile. (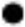) Adequate growth; (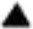), declining; (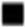), malnourished.

Figure 4 shows that early growth trajectories tracked into later childhood. The malnourished group was significantly smaller than the adequate growth group for all anthropometric measures as well as FMI and FFMI. The declining group was smaller than the adequate growth group in height-for-age Z score, BMIZ, mid-upper arm circumference, hip and waist circumferences but not in skinfolds, FMI or FFMI.

Clinical outcomes according to early growth trajectories are in Table 4. The declining group had lower systolic blood pressure than the adequate growth group but no other differences. The malnourished group had lower grip strength and blood TAG than the improving group and higher HbA1c and HDL-cholesterol. The prevalence of moderate and severe anaemia did not differ by early growth profile. There was a trend (*χ* ^2^ *P* = 0·08) towards differences in the proportion of HbA1c ≥ 6·5 % with 34 % of the adequate growth group, 40 % of the declining group and 58 % of the malnourished group having high HbA1c.

**Table 4.** Association of early growth latent variable classes with clinical variables using data from multiple imputation*,† (Odds ratios and 95 % confidence intervals)

	Imputed marginal means		Difference adjusted for age and sex			Multivariable coefficient		
Outcome	Means	95 % CI§	*β*	95 % CI§	*P* ‡	*β*	95 % CI||	*P* ‡
Grip strength (kg) adequate growth	22·6	21·7, 23·5	Ref			Ref		
Declining	21·7	20·9, 22·5	–0·84	–1·91, 0·23	0·12	–0·71	–1·79, 0·38	0·2
Malnourished	20·4	19·0, 21·7	–2·20	–3·72, −0·69	0·005	–1·87	–3·47, −0·27	0·02
Overall *P*			*P* = 0·02			*P* = 0·07		
Hb (g/l) adequate growth	104	100, 108	Ref			Ref		
Declining	107	103, 111	2·8	–3·0, 8·6	0·35	3·2	–2·8, 9·1	0·29
Malnourished	99	88, 109	–5·3	–16·4, 5·7	0·34	–5·2	–16·3, 5·9	0·36
Overall *P*			*P* = 0·30			*P* = 0·26		
HbA1c (%) adequate growth	5·7	5·5, 5·8	Ref			Ref		
Declining	5·9	5·6, 6·1	0·2	–0·1, 0·5	0·13	0·2	–0·1, 0·5	0·17
Malnourished	6·2	5·9, 6·6	0·6	0·2, 0·9	0·001	0·5	0·2, 0·9	0·005
Overall *P*			*P* = 0·004			*P* = 0·02		
Systolic blood pressure (mmHg)								
Adequate growth	107	105, 108	Ref			Ref		
Declining	104	102, 105	–2·9	–5·3, −0·5	0·02	–2·8	–5·2, −0·5	0·02
Malnourished	106	102, 110	–1·2	–5·5, 3·2	0·6	–1·8	–6·5, 2·9	0·46
Overall *P*			*P* = 0·05			*P* = 0·07		
Diastolic blood pressure (mmHg)								
Adequate growth	66	65, 67	Ref			Ref		
Declining	65	64, 67	–0·3	–2·0, 1·4	0·73	–0·4	–2·1, 1·3	0·62
Malnourished	65	63, 68	–0·6	–3·4, 2·3	0·69	–1·3	–4·3, 1·6	0·38
Overall *P*			*P* = 0·90			*P* = 0·67		
Blood lipids								
TAG (mmol/l) adequate growth	0·88	0·79, 0·97	Ref			Ref		
Declining	0·82	0·76, 0·88	–0·06	–0·17, 0·05	0·26	–0·04	–0·15, 0·07	0·45
Malnourished	0·63	0·55, 0·72	–0·25	–0·38, −0·12	< 0·001	–0·22	–0·37, −0·08	0·003
Overall *P*			*P* < 0·001			*P* = 0·003		
Cholesterol (mmol/l) adequate growth	3·84	3·69, 3·99	Ref			Ref		
Declining	3·84	3·72, 3·96	0·00	–0·19, 0·20	0·98	0·06	–0·14, 0·26	0·59
Malnourished	3·81	3·47, 4·15	–0·03	–0·40, 0·34	0·88	0·04	–0·34, 0·42	0·83
Overall *P*			*P* = 0·98			*P* = 0·86		
HDL cholesterol (mmol/l)								
Adequate growth	1·08	1·03, 1·13	Ref			Ref		
Declining	1·11	1·07, 1·15	0·03	–0·03, 0·09	0·38	0·03	–0·03, 0·10	0·35
Malnourished	1·29	1·15, 1·43	0·21	0·07, 0·36	0·005	0·23	0·08, 0·38	0·003
Overall *P*			*P* = 0·02			*P* = 0·01		
LDL cholesterol (mmol/l)								
Adequate growth	2·39	2·27, 2·51	Ref			Ref		
Declining	2·34	2·24, 2·44	–0·05	–0·21, 0·11	0·55	–0·01	–0·17, 0·15	0·94
Malnourished	2·19	1·91, 2·47	–0·20	–0·50, 0·10	0·2	–0·14	–0·45, 0·17	0·38
Overall *P*			*P* = 0·43			*P* = 0·64		

* HEU, HIV-exposed, uninfected; HUU, HIV-unexposed, uninfected.

† There were 112 children in the adequate growth class, 184 in the declining class and twenty-six in the malnourished class.

‡ *P* values within columns of coefficients are for the overall association with latent growth profile, while those in columns are for comparisons with the adequate growth group.

§ Marginal means controlling for age and sex; marginal means from multivariable differed from these only modestly and are not shown.

|| Multivariable coefficients represent the difference from the HUU group, adjusted for age, sex, maternal education, maternal marital status and socio-economic tercile.

### Combined HIV and early growth profiles

To explore evidence that early growth profile mediated the relationship between HIV exposure and outcomes, we selected outcomes associated, *P* < 0·05, with HIV exposure in age- and sex-controlled analyses (Tables 2 and 3): BMI, hip circumference, triceps and subscapular skinfolds, FMI, HDL and, because of its clinical importance and borderline associations, HbA1c. For all outcomes, the coefficients for HIV exposure became closer to zero and the significance decreased (online Supplementary Table S3). Coefficients for early growth classes changed little from those in age- and sex-controlled analyses shown in Fig. 4 and Table 4 (data not shown).

## Discussion

Similarly to when we studied the cohorts in 2014^(21)^, we found that by adolescence, HEU children had lower markers of adiposity than HUU children when values were adjusted only for age and sex. However, with further adjustment for socio-demographic factors, of which maternal education appeared to have the most important associations, these differences were no longer significant, suggesting that socio-demographic factors interacting with HIV exposure were important. Hb, HbA1c, blood pressure and most blood lipids also did not differ between HEU and HUU groups. HDL-cholesterol was higher in the HEU group. We wondered whether this was related to these children being thinner than the HUU children in this generally non-overweight population and found this was supported by exploratory analyses: when we included as covariables our indicators of adiposity (three skinfold thicknesses and FMI) the associations of HIV exposure with HDL were decreased and became non-significant (data not shown).

Perinatal HIV exposure may be associated with decreased *in utero* growth, as indicated by lower birth weight^(9,10)^, and the present study showed it was also associated in some children with having a malnourished early postnatal growth profile. Different early growth trajectories according to HIV exposure could result from catch-up growth or from ongoing environmental factors which may differ from those HUU children experience, for example, lower SES, increased exposure to infections in the household and increased risk of orphanhood. Exposure to parental opportunistic infections was likely common when the BFPH and CIGNIS children were young since ART was not generally available for their parents. Different early growth trajectories between HEU and HUU or in households with different socio-demographic characteristics could have influenced anthropometry, body composition and risk factors for chronic disease later in life. These early growth patterns tracked into adolescence with the groups remaining different in height and markers of both lean mass and adiposity. We used the adequate growth group as the reference in analyses but in general they exhibited low-risk markers for NCD, supporting previous studies which showed that early rapid growth is not a risk for NCD among children in low- or middle-income countries^(16,17)^. The children with the malnourished trajectory in early life had lower grip strength, higher HbA1c, lower blood TAG and higher HDL-cholesterol in early adolescence. Low grip strength is associated with adverse health outcomes in many adult populations^(31,32)^ and higher HbA1c suggests an increased risk of diabetes, though we recognise the limitations of the cut-off we used for high HbA1c. Poor postnatal growth has been linked with increased diabetes risk in high-income settings for men born early in the twentieth century^(33)^. Poor postnatal growth in the absence of specific diseases is currently rare in high-income countries but remains common in low- and middle-income countries. Our results differ from most studies of long-term health after childhood malnutrition which identify cases once they come to the clinic, whereas we were able to look at the critical infant period when children are still in the process of becoming malnourished.

Analysis of both HIV exposure and latent growth class reduced the associations between HIV exposure and outcomes which were significantly associated in the age- and sex-controlled analyses. This provides weak evidence that early growth mediates the relationship between maternal HIV exposure and outcomes. Further work is needed to determine the mechanisms whereby maternal HIV and socio-demographic variables led to poorer early growth and its consequences. We considered whether changes between initial recruitment in infancy and current follow-up in adolescence in the socio-demographic covariables investigated – mother’s education and marital status and SES – influenced our findings. SES was determined differently in the two original studies and at follow-up, so comparisons were not possible. Maternal education increased somewhat since recruitment so that the percentage with primary only decreased from 26 % to 14 %. More women at follow-up, 18 %, were widowed or divorced than in the original studies, 4 %; however, marital status was not associated with most outcomes. Furthermore, since we do not know at what stage these changes occurred, we were unable to control for them in analyses.

The study had several strengths including access to a well-characterised longitudinal cohort of children followed either from prenatal or infant life, detailed anthropometry, body composition measured in three ways and analysis using multiple imputation to account for missing data. An important limitation is the large loss to follow-up from both cohorts since their original recruitment up to 20 years before; however, we used a missing at random analysis and controlled in the multivariable regressions for recruitment variables which differed between participants who were followed up or not. Although we had detailed early growth data from both cohorts, the exact ages at which these were measured differed between cohorts and we had no birth lengths for CIGNIS children; both these factors could have affected the allocation of individuals to latent class resulting in the potential for misclassification bias. HbA1c levels were unexpectedly high in the cohort which made it difficult to determine a cut-off that would be associated with current or later diabetes. We considered whether there may have been technical problems with the instrument but we think this unlikely since a) the instrument was calibrated according to the manufacturer’s recommendations with both daily and weekly calibration, b) HbA1c values were not grossly elevated and c) the study was done in parallel with a related study in a slightly younger population and we did not find such high prevalence of HbA1c ≥ 6·5 % in those children^(28)^.

In conclusion, our results are encouraging by suggesting no serious long-term adverse effects on anthropometry, body composition or risk factors for NCD among most HEU children from a relatively middle-class urban Zambian population. However, parental HIV in some families may be associated with socio-demographic factors which lead to poor growth which tracks into adolescence and is associated with some NCD risk factors: low grip strength and high HbA1c. Our work is innovative in that it captured growth trajectory profiles over time when children were in the process of becoming malnourished, not just at a point when they were brought for care of severe malnutrition. More rapid growth during infancy and early childhood was not associated with increased NCD risk. Our results suggest social support for improved nutrition and better health care, not necessarily just for HIV-affected families although for them it could be delivered within ART support programmes, would benefit the long-term health of children in Zambia and other African settings.

## Supplementary material

For supplementary material accompanying this paper visit https://doi.org/10.1017/S0007114522001775. click here to view supplementary material
